# Developmental validation of Applied Biosystems YFiler Platinum Casework PCR Amplification Kit

**DOI:** 10.1038/s41598-023-41788-w

**Published:** 2023-09-04

**Authors:** Sumin Lee, Peterjon K. McAnany, Chien Wei Chang, Wilma Norona, Marc L. Short, Julio J. Mulero, Chang Zhong

**Affiliations:** Thermo Fisher Scientific Inc., 6065 Sunol Blvd, Pleasanton, CA 94566 USA

**Keywords:** Genotype, DNA, Biotechnology

## Abstract

The YFiler Platinum Casework PCR Amplification Kit is a 6-dye multiplex assay that simultaneously amplifies a set of 38 male-specific, Y-chromosome Short Tandem Repeat (YSTR) markers (DYS576, DYS389I, DYS635, DYS389II, DYS627, DYS549, DYS593, DYS645, DYS460, DYS458, DYS19, YGATAH4, DYS448, DYS391, DYS557, DYS522, DYS456, DYS390, DYS438, DYS392, DYS518, DYS444, DYS533, DYS570, DYS437, DYS385, DYS449, DYS643, DYS596, DYS393, DYS439, DYS481, DYF387S1, DYS527, DYS447), three insertion/deletion polymorphic markers (Yindels: rs771783753, rs759551978, rs199815934), and an internal quality control (IQC) system. When compared to the YFiler Platinum PCR Amplification kit for database samples, YFiler Platinum Casework kit was developed to include an improved Primer Mix incorporating a brighter TED dye, an updated internal quality control system, better resolution of large DNA fragments in Applied Biosystems POP-4 Polymer, and reduced female DNA cross-reactivity. Here, we report the results of the developmental validation study which followed the Scientific Working Group on DNA Analysis Methods (SWGDAM) guidelines and includes data for PCR-based studies, sensitivity, species specificity, stability, precision, reproducibility and repeatability, population concordance, stutter, DNA mixtures, and performance on mock casework samples. The results validate the multiplex design as well as demonstrate the kit’s robustness, reliability, and suitability as an assay for human identification with casework DNA samples.

## Introduction

Short tandem repeat (STR) analysis has become the gold standard for human identification and extends beyond the criminal justice system into areas including the identification of missing persons, mass disaster victims, parentage, and victims of human trafficking^1–3^. STR DNA analysis of the Y-chromosome (Y-STR analysis) allows for the identification of male-specific DNA markers for an additional level of discrimination. An increasing number of Y-chromosomal markers are needed for finer identification of male individuals and lineages, and the Chinese Ministry of Public Security (MPS) recently developed new standards for Y-STR loci when investigating male DNA to reduce the likelihood of adventitious matches to the Chinese National Database^4^.

In response to this recommendation, we developed the YFiler Platinum Casework kit, a 6-dye multiplex assay that simultaneously amplifies the same set of 38 male-specific, Y-STR markers found in the YFiler Platinum kit^5^. The YFiler Platinum Casework multiplex consists of 41 loci in a 6-dye configuration, that include all 27 YFiler Plus loci, 11 new Y-STR loci (DYS549, DYS593, DYS645, DYS557, DYS522, DYS444, DYS643, DYS596, DYS527a/b, DYS447), and three Yindels (rs771783753, rs759551978, rs199815934). Compared with its databasing companion kit, the YFiler Platinum Casework kit has been optimized for casework samples. The improvements include a redesigned internal quality control system, three redesigned Y insertion/deletion (Yindel) markers as smaller amplicons, improved resolution of larger loci in POP-4 Polymer, and a reduction of female DNA cross-reactivity. The YFiler Platinum Casework kit has also been validated for direct amplification of crude reference samples such as blood and buccal samples on (chemically treated or untreated) paper and swab substrates.

The developmental validation of the YFiler Platinum Casework kit described here was performed in accordance with the guidelines published by the Scientific Working Group for DNA Analysis Methods (SWGDAM)^6^ and regulations published by the China Ministry of Public Security^4,7^. The validation results demonstrate the YFiler Platinum Casework kit produces reliable and robust results making it suitable for forensic sample analysis.

## Materials and methods

### DNA samples

The 0.5 ng/µl male Control DNA 007 and 100 ng/µl female DNA 9947a were used in various validation experiments and were sourced from Thermo Fisher Scientific (Waltham, MA). Human blood samples from anonymous donors were purchased in the United States from blood bank Boca Biolistics (Coconut Creek, FL). All the samples used in the study were collected from healthy volunteer donors with self-reported ethnicities under Internal Review Board (IRB) approved informed consent. The DNA was extracted from blood samples using an ABI PRISM 6100 Nucleic Acid Prep Station (Thermo Fisher Scientific). Human genomic DNA, M15075, used for the degraded DNA experiments, was purchased from Coriell Institute (Camden, New Jersey). This DNA was subjected to heat degradation in boiling water under various durations to create different levels of degradation.

Species specificity studies were conducted using purified DNA. Genomic DNA of male primates (chimpanzee, gorilla, and orangutan) were purchased from BIOS Laboratories (New Haven, CT). Purified genomic DNAs from non-primate animal’s (cat, chicken, cow, dog, duck, fish, horse, mouse, pig, rat, and sheep) were obtained from Zyagen (San Diego, CA). Genomic DNAs from several human-associated microbial species (*Bacillus subtilis*, *Lactobacillus delbrueckii*, *Staphylococcus aureus*, *Escherichia coli*, and *Rothia mucilaginosa*) were obtained from the American Type Culture Collection (Manassas, VA) and pooled together. Microbiome DNA from soil was extracted from soil samples collected from the shores of the San Francisco Bay in California and extracted using the Invitrogen PureLink Microbiome DNA Purification Kit (Thermo Fisher Scientific).

Quantification of human genomic DNA was carried out using the Applied Biosystems Quantifiler Trio DNA Quantification Kit using the Applied Biosystems 7500 Real-Time PCR System. Concentration of non-human DNA was determined by measuring the absorbance of the sample at 260 nm.

### PCR primer set and master mix components

The YFiler Platinum Casework Kit includes 41 loci (38 Y-STRs and 3 Y-Indel) in a 6-dye, J6-T configuration (6-FAM, VIC, TED, TAZ, SID, and the LIZ for size standard). Additionally, it includes two Internal Quality Control (IQC) markers (IQCS and IQCL) that bracket the read region within the FAM channel. The primers for the two IQC markers amplify synthetic DNA targets included in the primer mix. IQCS is a low molecular weight amplicon, 60 nt in length. Whereas IQCL is a high molecular weight amplicon, 583 nt in length.

Developed as the companion kit for the YFiler Platinum kit, the YFiler Platinum Casework kit contains the same loci as those in the YFiler Platinum kit. To maximize the genotype concordance, most of the YFiler Platinum Casework primer sets have the identical nucleotide sequences to those in the YFiler Platinum kit. Among DYS557, DYS456, DYS437, and DYS533, at least one primer has the same nucleotide sequence as in YFiler Platinum kit, but other primers for the locus were redesigned to adjust the size of the amplicon and increase male specificity. There are newly designed primer sets included in the YFiler Platinum Casework kit for DYS645, DYS19, DYS522, DYS444, DYS596, DYS527, DYS447, rs771783753, rs759551978, and rs199815934, which are not found in YFiler Platinum nor the YFiler Plus kits.

The PCR Master Mix components of the YFiler Platinum Casework kit include a “hot-start” Taq DNA polymerase, buffer, salts, dNTPs, detergent, carrier protein, sodium azide, and stabilizing components. Master Mix components were optimized for robust and sensitive DNA detection, as well as high male specificity. The performance was evaluated by varying individual components in 10% increments out to + / − 20% (v/v) from the standard formulation to confirm the reliability and robustness of the formulation. Three sample types include 1 ng male 007 DNA, 1 µg female 9947A, and 1 ng male 007 DNA in the presence of 100 ng/µL of humic acid PCR inhibitor, were evaluated in four replicate PCRs for each component at each concentration.

### PCR amplification and thermal cycling conditions

The YFiler Platinum Casework kit amplification reactions has been optimized in a 25 μL total volume, consisting of 7.5 μL of Master Mix, 7.5 μL of Primer Mix and 10 μL of sample input. Samples were amplified in Applied Biosystems MicroAmp Optical 96-well reaction plates (Thermo Fisher Scientific).

Standard thermal cycling conditions for the kit are: enzyme activation at 95 °C for 1 min; 29 cycles of denaturation at 94 °C for 4 s and annealing ⁄ extension at 61 °C for 60 s; and a final extension step at 60 °C for 8 min. Thermal cycling was performed on the Applied Biosystems ProFlex or VeritiPro thermal cyclers with standard 96-well block, or on the GeneAmp PCR system 9700 with a gold-plated silver block. Ramp rates used for the different thermal cyclers were: “9700 Simulation” mode for the ProFlex, “9700 Max” mode for the VeritiPro, and “Max” mode for the 9700. The YFiler Plus Kit (Thermo Fisher Scientific) used for benchmarking studies was run under standard conditions^8^.

### Sample electrophoresis and data analysis

Separation of the PCR amplicons was performed on the Applied Biosystems 3500xL Genetic Analyzer with Data Collection software 4.0.1 using J6-T dye set. Performance checks were also done on the Applied Biosystems SeqStudio (4-capillary), 3500 (8-capillary) and the 3130xl (16-capillary) Genetic Analyzers.

PCR amplicons were prepared for capillary electrophoresis (CE) by adding 1 μL of the PCR product (or Allelic Ladder) to 10 μL of formamide/size standard solution (9.6 μL of deionized Hi-Di™ Formamide plus 0.4 μL of Applied Biosystems GeneScan 600 LIZ Size Standard v2.0; Thermo Fisher Scientific). Samples were denatured at 95 °C for 3 min then immediately chilled on ice prior to electrophoresis. Samples analyzed on the 3500xL instrument were injected at 1.5 kV for 24 s and electrophoresed at 13 kV for 1600 s in POP-4 Polymer (Thermo Fisher Scientific). Runs on the 3500 instrument (8-capillary) used similar electrophoresis conditions, except the injection time was reduced to 16 s. Samples analyzed on the 3130xl instrument were injected at 3 kV for 8 s, with electrophoresis at 14 kV for 1800s. Samples analyzed on the SeqStudio instrument were injected at 1.2 kV for 10 s, with electrophoresis at 11 kV for 1120 s.

Data analysis was performed using Applied Biosystems GeneMapper *ID-X* Software v 1.6 (Thermo Fisher Scientific) with a peak amplitude threshold (PAT) at 175 relative fluorescence units (RFU) and an analysis region of 60–540 nt.

### Species specificity

The YFiler Platinum Casework kit primers were designed to be human-specific with minimal cross-reaction with non-primate animals or microbial species. The primers were thoroughly in silico searched against various DNA databases from human and microbial genomes. 1 ng each of non-primate DNAs (section "DNA samples"), primate DNA, pooled oral microorganism DNAs, and 20 ng extract of soil microbiome DNAs were amplified in duplicates. All amplifications were conducted at 29 PCR cycles then electrophoresed on the 3500XL instrument and the data analyzed for above-background peaks in or near the read region.

### Inhibition models

YFiler Platinum Casework kit performance was evaluated in the presence of PCR inhibitors such as hematin, humic acid, and tannic acid^9,10^. Stock solutions of each inhibitor compound were prepared as follows: hematin (Sigma, St. Louis, MO) was dissolved in 0.1 N NaOH; humic acid (Alfa Aesar) and tannic acid (Fisher Scientific) were dissolved in molecular biology grade distilled water (Ambion). Test samples were prepared with a constant input of 1 ng of 007 DNA per reaction and increasing concentrations of each inhibitor: 200 and 400 µM hematin, 100 and 200 ng/µL of humic acid, or 100, and 300 ng/µL of tannic acid (all final concentrations in PCR). The Applied Biosystems YFiler Plus PCR Amplification Kit was used for comparison.

### Degraded DNA

Degraded human male genomic DNA was prepared by heating M15075 DNA samples at 95 °C for 15 and 45 min to create “low” and “high” degraded DNA samples respectively^11^. To evaluate YFiler Platinum Casework kit performance against degraded DNA, 1 ng of these degraded samples and an undegraded control were amplified with four replicate PCRs per sample. Performance measured as allele recovery was benchmarked against that of the YFiler Plus assay.

### Sensitivity study

Serial dilutions of control DNA 007 were prepared as following in total PCR reaction: 1000, 500, 250, 125, 62.5, 31.2 and 15.6 pg DNA. Non-template controls (NTCs) were run in parallel. Samples were amplified with both YFiler Platinum Casework and YFiler Plus kits.

### Male specificity

The male specificity of the YFiler Platinum Casework kit was tested with a wide range of male and female DNA mixture samples. The ratio between male and female DNA were varied as follows: 1:1000, 1:2000, 1:4000, 1:8000, and 1:16,000 (all with 1 µg of female DNA) as well as 1:24,000, and 1:48,000 (both with 3 µg female DNA). Replicates of four were tested, and the amplified products were analyzed for cross-reactive artifacts in or around the read region, as well as male allele recovery.

### DNA mixtures

DNA from two human cell lines (M007 Control DNA and M15075) were combined in 1 ng total input but different mixture ratios: 1:1, 1:3, 1:7, 1:15, 1:30 (i.e. 1:30 is 32 pg and 968 pg = 1000 pg). Each sample was run in 4 replicate reactions with the YFiler Platinum Casework and YFiler Plus kits and the number of non-overlapping alleles between the kits were counted.

### Sizing accuracy, precision, and stutter ratio calculation

Allele sizing and accuracy studies were carried out using the following Genetic Analyzers: 3500xL, 3500, 3130xL, and SeqStudio systems. Genomic DNAs from 42 randomly selected male donors were amplified at 1 ng total DNA with the YFiler Platinum Casework kit. The sizing accuracy was calculated using the size difference between the allele peak with the corresponding allele in the allelic ladder. Sizing precision was evaluated by allele size standard deviations of repetitive injections of the YFiler Platinum Casework allelic ladder. Stutter ratios for the YFiler Platinum Casework were calculated using the peak height ratio of the stutter peak and corresponding allele peak. Genomic DNAs from 356 in-house samples were used in the stutter calculations. The samples were run on an 3500xL Genetic Analyzer using a minimum stutter peak height of 20 RFU for stutter calculation. All types of stutter peaks were included: minus stutters (the most significant form), plus stutters, and in some cases partial-repeat stutters with 2-bp spacing (e.g. DYS19, DYS596).

### Population and concordance studies

The genotypes of 484 unrelated individuals with self-declared Chinese ethnicity were determined using the YFiler Platinum Casework Kit and the YFiler Platinum Kit under standard conditions. Genotype concordance was compared between these two kits. Gene Diversity values for each locus were calculated. The same set of population samples were also genotyped using the YFiler and YFiler Plus kits using standard conditions. The results were used to compare Haplotype Diversity and Discrimination Capacity values among all kits. All the human samples used in the study were purchased from blood bank Boca Biolistics (Coconut Creek, FL), and were collected from healthy volunteer donors with self-reported ethnicities. All the participants or their legal representatives provided written informed consent. No live animals were used in this study. The Thermo Fisher Scientific Internal Review Board approved the study protocols and granted ethical clearance for the studies performed in this validation (PCP0053079). The study followed The Scientific Working Group on DNA Analysis Methods (SWGDAM) guidelines^6^.

### Statistical analysis and color balance definitions

In the case of multi-copy loci such as DYS385, DYS387S1, and DYS527, the peak heights were determined by averaging the heights of two peaks. To assess the intracolor balance (ICB), the lowest peak height within each dye channel was divided by the highest peak height within the same dye channel. The resulting value was reported as a percentage. The statistical analysis of the data was performed using Minitab (Minitab Inc., State College, PA) or JMP (SAS Institute Inc., Cary, NC) software.

## Results and discussion

The experimental methods and descriptions in this study follow the format of previous validation studies for other PCR amplification kits created by our team^12,13^.

### Primer mix and master mix

**Figure 1:**
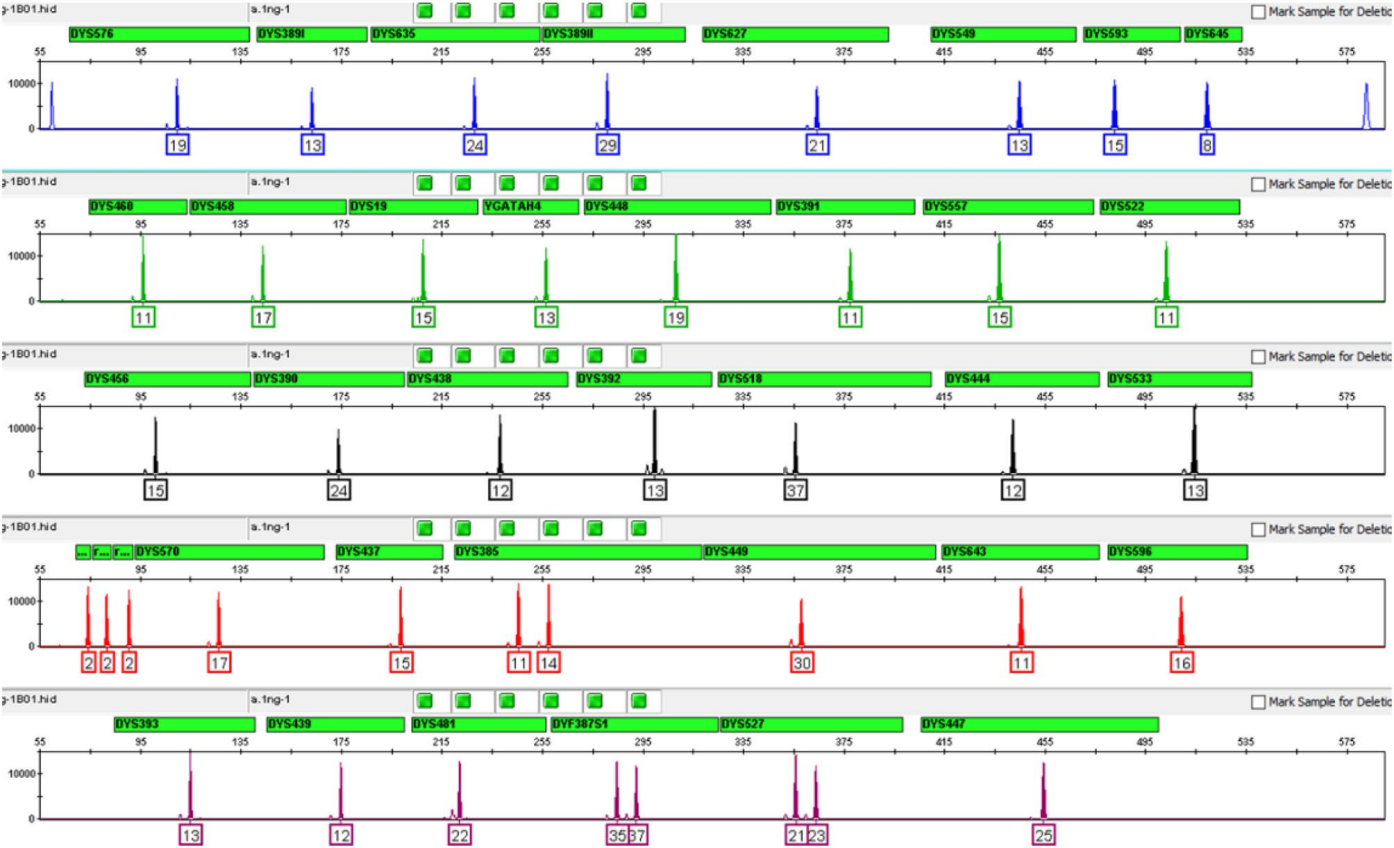
Representative profile generated from 1 ng of Control DNA 007 amplified with the YFiler Platinum Casework kit for 29 cycles and electrophoresed on an Applied Biosystems 3500xL Genetic Analyzer. Y axis: 15,000 RFU.

The YFiler Platinum Casework kit brings about meaningful changes that differentiate it from the YFiler Platinum kit for database samples. The YFiler Platinum Casework kit features a J6-T dye set configuration utilizing a brighter TED dye to replace the NED dye in the YFiler Platinum kit to improve detection of low input samples. The primers of the three Y-Indel markers were redesigned to significantly reduce their size from above 500 nt to below 90 nt. This mini design improves the detection of these markers in highly degraded samples. The multi-copy DYS527 primers were redesigned to reduce the amplicon sizes by approximately 150 nt when compared to the original YFiler Platinum design. This improves 1 bp resolution of microvariants under POP4 CE condition. Besides these size changes, some markers (e.g. DYS596, DYS645) were redesigned to minimize female DNA cross-reactivity. A typical profile for 1 ng of M007 control DNA is shown in Fig. 1. The two blue peaks flanking the read region at 60 nt and 583 nt in the blue (FAM) channel are the IQC system peaks, used to monitor PCR quality (section "Inhibited and degraded DNA").

Complete STR profiles were successfully recovered when the concentrations of primer sets deviated by ± 20% from the recommended values, whether in pristine conditions or in the presence of 100 ng/μl humic acid. Although there was a slight decrease in peak heights when primer mix concentrations were lower, no significant impact on intracolor peak height balances was observed. Further investigations were conducted on specific components of the master mix. It was discovered that potassium and magnesium concentrations had the most significant influence on peak heights and peak balance. Despite variations of ± 20% in magnesium and potassium levels, full profiles were obtained for all tested concentrations. However, lower magnesium or potassium levels resulted in lower allele peak heights and poorer overall peak balance^14^. The reduced overall peak balance was primarily caused by increased peak heights of DYS447 in comparison to other loci. Magnesium and enzyme concentrations also affected the male specificity of the assay. Higher concentrations of magnesium and enzyme led to an increase in cross-reactivity peaks for females^14^. Overall, reagent concentrations in the master mix were optimized to provide maximal assay sensitivity and color balance while maintaining male specificity.

### Thermal cycling optimization

The YFiler Platinum Casework kit’s thermal cycling conditions were optimized to yield minimal non-specific background, enhanced sensitivity and robustness, and maximal male specificity. To study the robustness of this protocol, a range of times and temperatures were tested for each phase of amplification: denaturation, annealing/extension, and final extension hold.

The YFiler Platinum Casework kit standard thermal cycling protocol is described in 2.3. Validation experiments were performed as guard band studies, with test settings incrementally varied around the standard time and temperature settings. The following ranges of test settings were used in the study (standard settings in bold): Amplification cycle number (27, 28, **29**, 30, and 31 cycles) Denaturing temperature (92, 93, **94**, 95, and 96 °C), Anneal/Extend temperature (59, 60, **61**, 62, and 63 °C), and Final Extension Time (2, 5, **8**, 11, 14 min). After capillary electrophoresis, the results were assessed for standard performance metrics such as ICB in every channel.

For the cycle number test, an increase in cycle number correlated with an increase in overall peak height while maintaining peak balance^14^. A value of 29 cycles was chosen to balance maximal sensitivity with minimal noise and off-scale peaks. Compared with the YFiler Plus 30 cycle protocol, one less cycle number was needed in the YFiler Platinum Casework Kit due to the brighter TED dye used. Varying denaturing temperature +/− 2 °C from the optimal 94 °C did not show any noticeable change in peak heights and overall balance. In contrast, the annealing/extension temperatures had a significant impact in performance. Higher temperatures improved male specificity; but reduced peak heights for several markers. At 63 °C, peak height from multiple markers, including DYS460, DYS456, DYS390, and DYS439, dropped significantly^14^. The final extension time was used to drive the completion of the non-templated adenosine addition^15^. Results from varying final extension time over a range of 2–14 min showed no “split” or “shoulder” peaks from “+A” addition^14^.

### Species specificity

The species specificity study was performed to demonstrate that cross-reactivity with non-human DNA is minimal and does not interfere with the kit’s ability to return reliable results from human samples. When challenged with a range of animal and microbial DNA templates, amplification was observed only with 1 ng of chimpanzee DNA, which generated a partial STR profile with a majority of the thirty-four allelic peaks detected as off-ladder^14^. While the gorilla DNA yielded reproducible heterozygous off-ladder SID-dye labeled peaks at 123.60 and 131.55 bp^14^. This is not unexpected as high degree of gene homology between humans and primates, and these artifacts were also reported in other STR kits^12,13^. Among the non-primate species, no reproducible cross-reactivity peaks were observed above a threshold of 175 RFU. All the tested species (mouse, dog, pig, horse, cat, rat, chicken, duck, sheep, cow, and fish, and pooled oral microorganisms), and 20 ng extract of soil microbial population exhibited no reproducible amplification products above 175 RFU.

### Sensitivity

**Figure 2:**
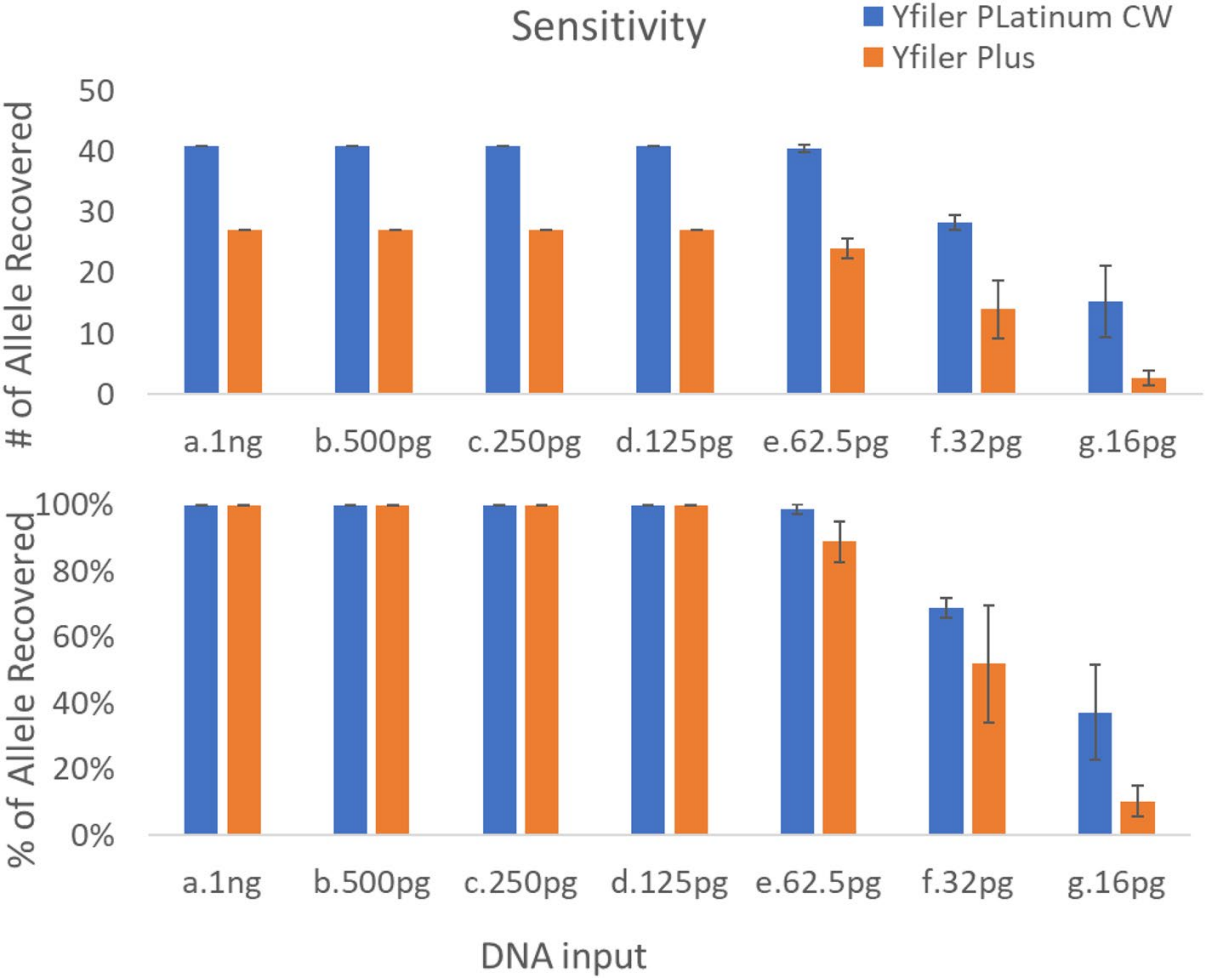
Results of a sensitivity study using a serial dilution of 007 control DNA. Full STR profiles were obtained with 125 pg input. At lower sample inputs, allelic drop-out events were detected. The blue bar represents the YFiler Platinum Casework kit (full profile = 41 alleles) and the orange bar represents the YFiler Plus kit (full profile = 27 alleles).

The sensitivity assessment of the YFiler Platinum Casework kit involved the utilization of control M007 DNA. Replicates of four were tested with a range of DNA inputs, varying from 1 to 16 pg, along with Non-Template Control (NTC). The study also included the YFiler Plus kit for benchmarking purposes. To mitigate potential variations in signal intensity arising from different instruments, all amplicon separation and detection procedures were carried out on the same 3500xL instrument. Full profiles were obtained consistently at 125 pg input or greater with both chemistries (Fig. 2). At 62.5 pg, the YFiler Platinum Plus kit showed that 2 out of 4 replicates gave a full profile compared to YFiler Plus kit showing several dropouts in all replicates. All things being equal, the YFiler Platinum casework kit yields more alleles than the YFiler Plus kit because it has more markers. For the NTC, no background noise or extraneous peaks were observed above 100 RFU in the read region for any of the four replicates^14^.

### Inhibited and degraded DNA

Forensic casework samples have the potential to harbor chemical contaminants that may compromise the PCR reaction. Validation studies often incorporate models of PCR inhibition to evaluate the resilience of the assay. Hematin, humic acid, and tannic acid serve as representative models for environmental contamination from blood, soil, and leather^9,10^. In these experiments, mock inhibition samples were prepared by spiking a fixed concentration of human male control DNA 007 (1 ng total per PCR reaction) with increasing concentrations of PCR inhibitors.

**Figure 3:**
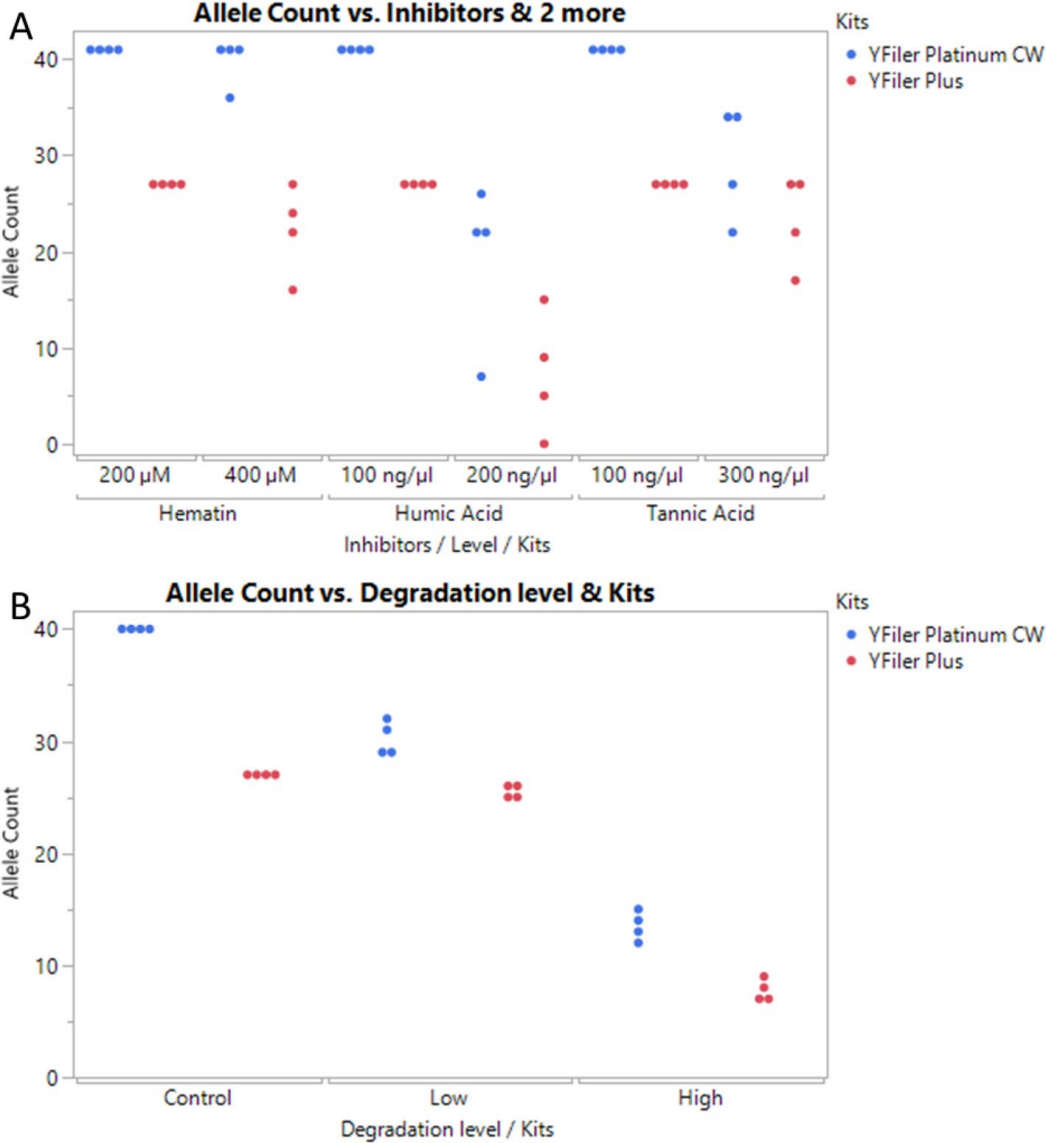
Allele counts per replicate PCR in the presence of inhibitors and degraded samples are shown for the YFiler Platinum Casework and YFiler Plus kits. (**A**) Three PCR inhibitors at two concentrations: 100 and 200 ng/µL humic acid, or 200 and 400 µM hematin, or 100 and 300 ng/µL tannic acid. (**B**) Two levels of DNA degradation were tested: Low degraded, and highly degraded human genomic DNAs.

The YFiler Platinum Casework kit was compared to the YFiler Plus kit with inhibited samples (Fig. 3A) using as a metric the alleles recovered from each kit. Full STR profiles (41 alleles for 007 DNA) were obtained for the lower levels of inhibitor, and partial profiles were obtained with the highest levels of inhibitors (400 µM hematin, 200 ng/µL humic acid, and 300 ng/µL tannic acid). In all conditions, the YFiler Platinum Casework Kit recovered more alleles than YFiler Plus Kit.

Another common challenge encountered in DNA casework analysis is the degradation of sample DNA. These samples have been exposed to adverse environmental conditions such as sunlight, chemical substances, or microbial enzymes. This degradation leads to a "ski slope effect" profile during capillary electrophoresis, as longer DNA templates are more susceptible to degradation than shorter ones. Figure 3B displays the allele counts obtained with a model of degraded DNA. Full STR profiles (40 alleles) were obtained for the "Control" fractions, while allele dropouts were observed in both the "Low" and "High" degradation fractions. Notably, the redesigned Y indel alleles were recovered in the highly degraded samples, highlighting the significance of these mini-loci. As with the previous study, the YFiler Platinum Casework Kit consistently yielded more alleles than the YFiler Plus Kit in the degraded sample study.

**Figure 4:**
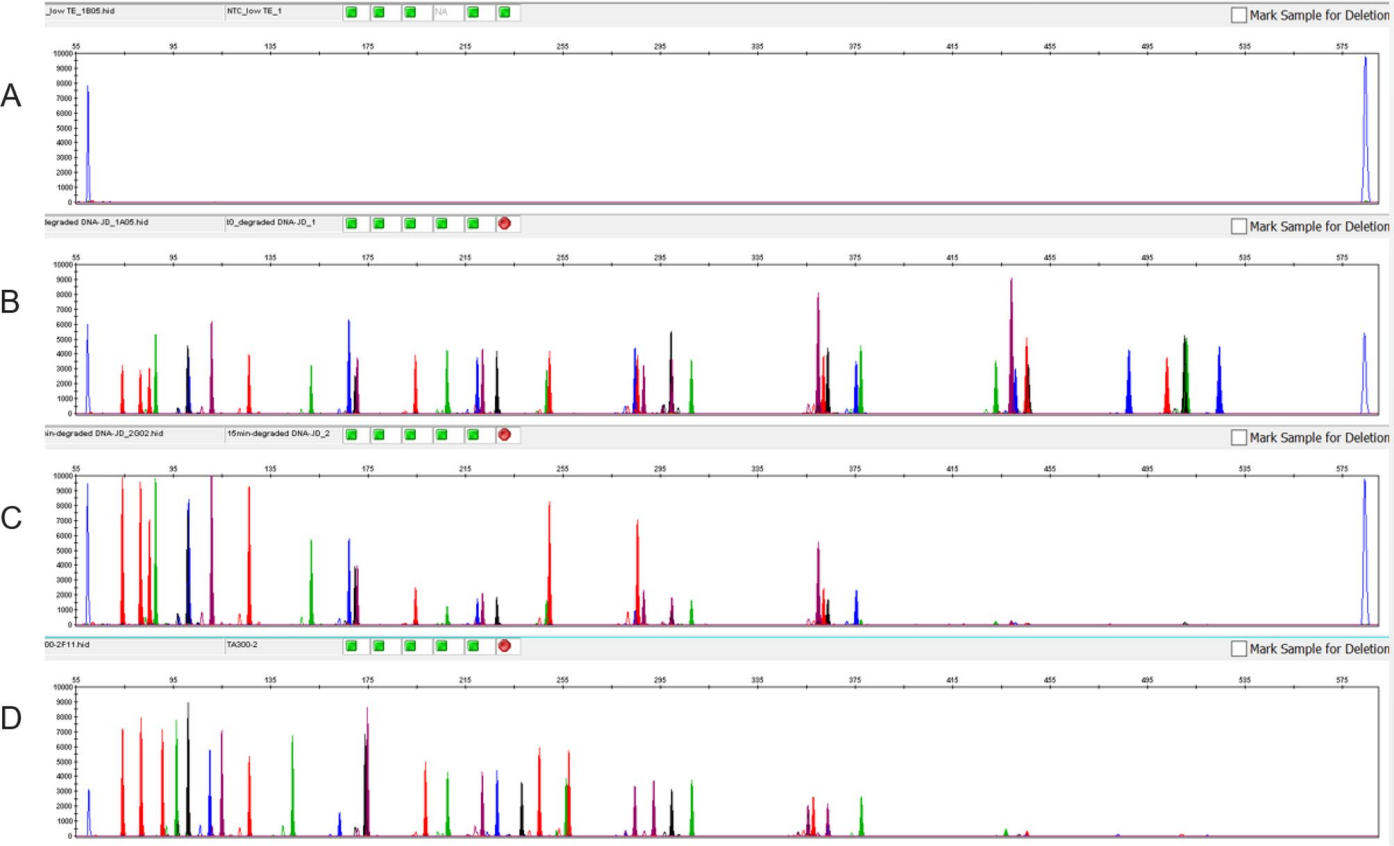
IQC system and interpretation. (**A**) Negative sample with no human genomic DNA, will show balanced IQCS and IQCL peaks; (**B**) positive sample (M007 1 ng) showing a balanced profile of STR alleles and IQC peaks; (**C**) degraded DNA with ski slope profile and balanced IQC peaks; (**D**) inhibited sample (tannic acid at 300 ng/µL) with ski slope profile but with decreased IQCL peak height.

The YFiler Platinum Casework includes an Internal Quality Control (IQC) system to assess the efficiency of the PCR reaction. Combined with the overall peak heights of the STR amplification products, the IQC can indicate whether a sample shows signs of degradation or inhibition. Under optimal PCR conditions, the peak height of the IQCL is approximately equal to or slightly higher than the IQCS (Fig. 4A,B). If the profile displays a ski slope with balanced IQC peaks, it indicates that PCR has occurred optimally, but the sample may be degraded (Fig. 4C). Under suboptimal PCR conditions, such as inhibition, the height of the IQCL is significantly reduced relative to the IQCS (Fig. 4D).

From a practical standpoint, if the sample is found to be inhibited, a second amplification reaction with re-diluted sample may improve the profile by generating more alleles. If the sample is degraded, higher sample input may increase the chance of detecting larger amplicons over the detection threshold.

### Male specificity studies

SWGDAM^16^ recommends the use of Y-STRs for processing male DNA in mixtures that contain an excessive amount of female DNA. To attain this goal, it is critical to minimize female cross-reactivity in the read region. To evaluate the ability of YFiler Platinum Casework kit to detect male DNA in a high female DNA background, we created mixture samples by combining 007 control DNA (male) and 9947A DNA (female) at different ratios. The ratios were: 1:1000, 1:2000, 1:4000, 1:8000, 1:16,000, where female DNA remained at 1 µg and male DNA varied from 1 ng to 62 pg; and 1:24,000, and 1:48,000, where female DNA remained at 3 µg and male DNA varied from 125 to 62 pg. None of these samples generated female artifacts above a PAT of 175 RFU^14^.

**Figure 5:**
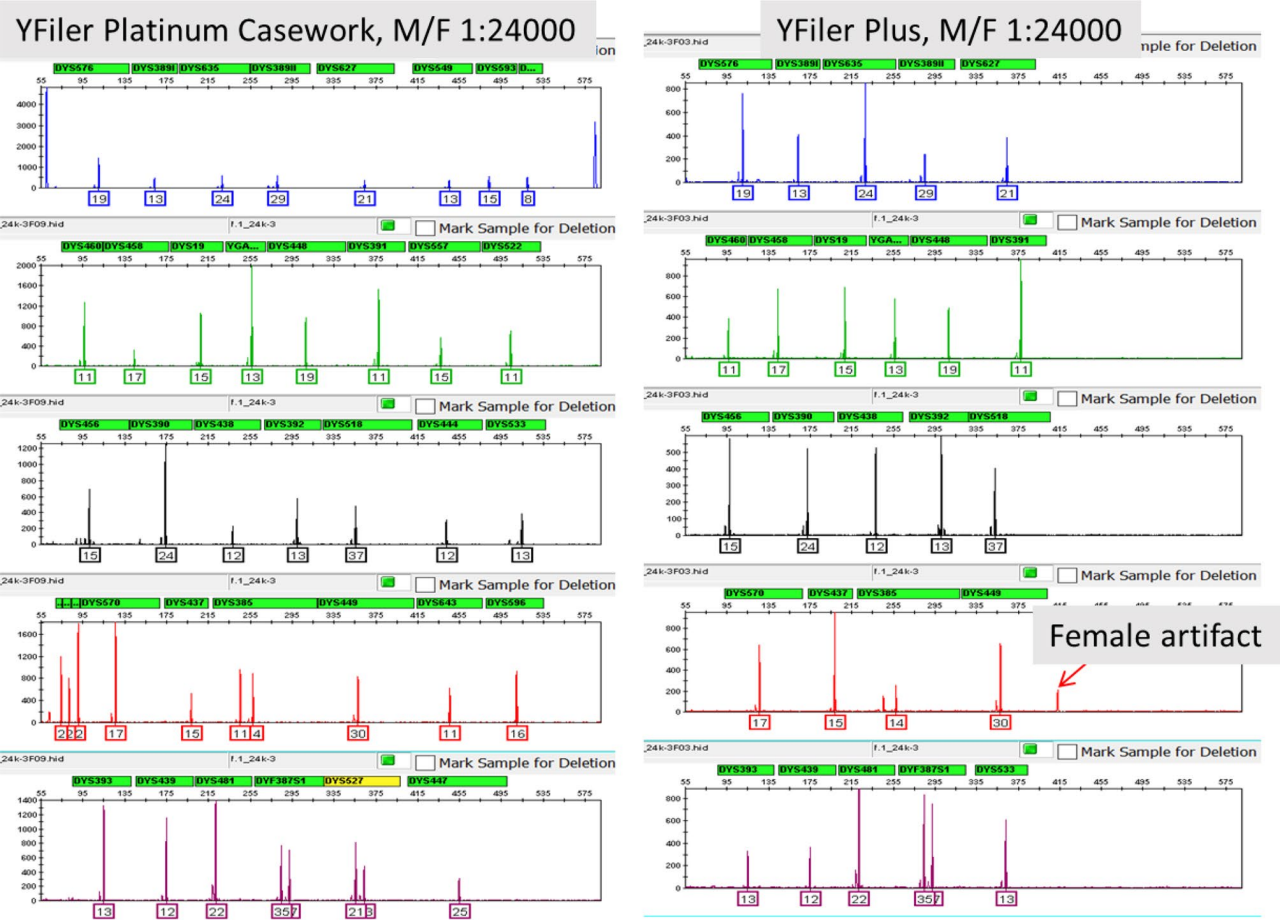
Full male DNA profile obtained with the YFiler Platinum Casework kit in a 1:24,000 male/female mixture. For comparison, the YFiler Plus DNA profile yielded a full profile but it had an extra peak (female artifact) in the red channel outside of the region of interpretation.

Figure 5 shows the comparison profiles of YFiler Platinum Casework kit with YFiler Plus kit with the 1:24,000 male/female mixture. Full profiles were obtained from YFiler Platinum Casework kit while one replicate of YFiler Plus kit produced one dropout. In addition, YFiler Plus recovered a significantly smaller number of alleles due to having less markers. Besides generating a full profile, the YFiler Platinum Casework kit did not show reproducible artifacts above 175 RFU in the presence of female DNA inputs of 3 µg. However, the YFiler Plus Kit produced a reproducible known artifact at 412 bp in the TAZ channel^13^ crossing the 175 RFU peak amplitude threshold. This artifact did not affect profile interpretation because it lies outside the read region (Fig. 5).

### DNA mixture studies

In forensic casework samples, it is common to encounter male/male DNA mixtures. It is important for the Y-STR multiplex assay to accurately distinguish the minor contributor from the major contributor. To assess the YFiler Platinum Casework kit's ability to resolve alleles from minor contributors, we established a mixture model system using selected male genomic DNAs. The goal was to maximize the presence of unique, non-overlapping alleles. Non-overlapping alleles are those that are not present in the other contributor, nor in the plus- or minus-stutter positions of alleles present in the other contributor. Two male DNAs, M007 Control DNA and M15075, were prepared at ratios ranging from 1:1 to 1:30 with M15075 as the minor contributor and a total DNA input of 1 ng. Both the YFiler Platinum Casework and YFiler Plus kits were used to amplify mixed samples and the number of non-overlapping STR alleles were counted. (Table 1). The mixture sample generates 13 non-overlapping alleles for the YFiler Platinum Casework kit and 10 for the YFiler Plus kit. All the non-overlapping alleles from the minor contributor were recovered with as low as 125 pg DNA from the minor contributor (1:7 ratio).

**Table 1:** Mixture of two male DNAs.

Male/male ratio	YFiler Platinum Casework				YFiler Plus			
1:1	**13**	**13**	**13**	**13**	**10**	**10**	**10**	**10**
1:3	**13**	**13**	**13**	**13**	**10**	**10**	**10**	**10**
1:7	**13**	**13**	**13**	**13**	**10**	**10**	**10**	**10**
1:15	**13**	**13**	**13**	12	**10**	**10**	**10**	9
1:30	5	5	6	5	4	4	6	5

The table shows mixture ratios from 1:1 to 1:30 (M15075:M007) and non-overlapping allele counts per reaction (N = 4 replicate reactions per mixture sample) for YFiler Platinum Casework and YFiler Plus kits. The recovery of all possible non-overlapping alleles was highlighted in bold.

### Stutter products

Stutter products are common artifacts of the PCR amplification as a result of strand slippage^17,18^. These artifacts can complicate interpretation, therefore are usually filtered-out by genotyping analysis software such as GeneMapper ID-X using imported marker-specific stutter filters. These filter values were empirically determined for each STR locus by analyzing YFiler Platinum Casework assay results from a population study of 356 male genomic DNAs. For each locus, stutter ratios for all observed alleles in the population samples were determined as the stutter peak height divided by its corresponding true allele peak height.

The ratios of all stutters for the YFiler Platinum Casework kit markers are shown in Table 2. The range of observed stutter ratios had non-normal distributions for most STR loci. Marker-specific stutter filters were set using fitted distributions of stutter data so that approximately 99.75% of stutters would be filtered-out during genotyping analysis. The remaining 0.25% of non-filtered stutters were deemed to be statistical outliers. As observed with other STR multiplexes, pentanucleotide (e.g. DYS593, DYS438) and hexanucleotide loci (e.g. DYS596) had some of the lowest minus-stutter values while the lone trinucleotide repeat marker DYS481 had the highest. Partial-repeat stutters (e.g. 2-bp spacing) were reproducibly observed in DYS19 and DYS596 and stutter ratios were provided to filter out these artifacts.

**Table 2:** YFiler Platinum Casework kit stutter summary.

Marker	Repeats	− Stutter	+ Stutter	Marker	Repeats	− Stutter	+ Stutter
DYS576	4	0.130	0.062	DYS456	4	0.144	0.066
DYS389I	4	0.082	0.033	DYS390	4	0.116	0.018
DYS635	4	0.112	0.044	DYS438	5	0.040	0.013
DYS389II	4	0.155	0.084	DYS392	3	0.148	0.092
DYS627	4	0.134	0.039	DYS518	4	0.210	0.060
DYS627 (− 2 bp)	4	0.021	–	DYS444	4	0.086	0.031
DYS549	4	0.103	0.060	DYS533	4	0.091	0.021
DYS593	5	0.013	0.014	DYS570	4	0.133	0.034
DYS645	5	0.024	0.010	DYS437	4	0.068	0.017
DYS460	4	0.095	0.034	DYS437 (− 5 bp)	4	0.026	–
DYS458	4	0.143	0.023	DYS385	4	0.154	–
DYS19 (− 4 bp)	4	0.097	0.029	DYS449	4	0.198	0.026
DYS19 (− 2 bp)	4	0.086	–	DYS643	5	0.054	0.053
YGATAH4	4	0.089	0.019	DYS596	6	0.024	0.039
DYS448	6	0.035	0.017	DYS596 (− 2 bp)	6	0.023	0.009
DYS391	4	0.083	0.028	DYS393	4	0.124	–
DYS391 (− 5 bp)	4	0.023	–	DYS439	4	0.089	0.044
DYS391 (− 10 bp)	4	0.018	–	DYS481	3	0.256	0.052
DYS557	4	0.141	0.042	DYS481 (− 2 bp)	3	0.077	0.063
DYS522	4	0.085	0.031	DYS481 (− 2 bp)	3	0.070	0.045
DYS522 (− 2 bp)	4	0.009	–	DYS481 (− 6 bp)	4	0.069	–
				DYF387S1	4	0.128	–
				DYS527	4	0.125	0.039
				DYS447	5	0.052	0.044

The YFiler Platinum Casework kit stutter filter settings were based on fitted distributions of stutter observed for each marker in a population study of 356 individuals.

### Sizing precision and accuracy

**Figure 6:**
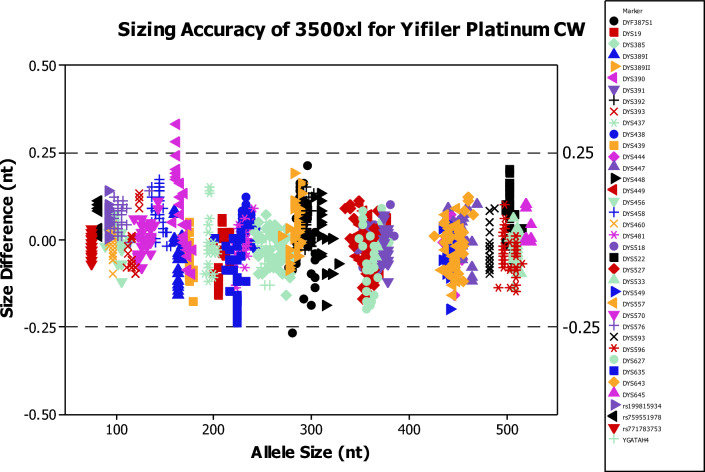
Allele size (nt) versus corresponding allelic ladder allele size (nt) for 42 samples processed on the 3500xL genetic analyzer. All the size deviations are within the 0.5 nt range that is required for accurate genotyping.

Sizing precision and accuracy measurements were performed on Applied Biosystems SeqStudio, 3130xl, 3500, and 3500xL CE platforms. The sizing precision was evaluated by injecting allelic ladder in all capillaries repeatedly, followed by calculating the mean and standard deviation of the size for each allele. All alleles demonstrated an ideal size deviation of less than 0.15 nt. The sizing accuracy was calculated by the size deviations of allele peaks to their corresponding allele in the allelic ladder. A total of 42 population samples were analyzed on various CE instruments. Figure 6 shows the sizing accuracy on 3500xL. Similarly, the alleles in all samples run on the SeqStudio, 3130xl, and 3500 CE platforms met the size deviations within ± 0.5 nt^14^. These results demonstrate that the YFiler Plantium Casework kit has sufficient precision and accuracy to resolve 1 nt differences required for correct genotyping.

### Population studies and genotype concordance

The YFiler Platinum Casework kit was designed to meet the rigorous requirements of the Chinese forensic laboratories. For this reason, population testing was conducted with 484 presumably un-related Chinese individuals. Each sample was genotyped with the YFiler Platinum Casework kit, as well as its counterpart databasing kit, the YFiler Platinum kit. Genotype concordance of 100% was achieved between these two kits. Gene Diversity values for each YFiler Platinum Casework locus in the Chinese population was also calculated ^14^.

Compared with earlier generation YFiler and YFiler Plus kits, the YFiler Platinum Casework kit contains more loci, and thus has a higher discrimination power. To demonstrate this increased capability, YFiler and YFiler Plus kits marker sets were compared using the same set of population samples. Discrimination Capacity (DC) and the number of unique haplotypes (UH) for these three different Y-STR kits are presented in Table 3. The results from Table 3 showed that the differences in DC and UH between YFiler Plus and YFiler Platinum are very small when compared to the differences observed for both assays against YFiler.

**Table 3:** Discrimination capacity (DC) and unique haplotypes (UH) of the YFiler Platinum Casework kit and other Y-STR kits from a population of 484 presumed non-related individuals.

Kit	No. of STR Loci	DC (%)	UH
YFiler	17	92.36	447
YFiler Plus	27	95.25	461
YFiler Platinum Casework	41	95.66	463

## Conclusion

The YFiler Platinum Casework PCR Amplification Kit was designed and optimized for the processing of casework samples and meets Chinese MPS recommendations for male-specific DNA testing of forensic samples. The 41-plex amplification kit simultaneously amplifies the same set of 38 male-specific Y-STR loci, and three insertion/deletion polymorphic markers (Y-indels), found in the YFiler Platinum Kit for database samples but it has been optimized for casework samples. The increased multiplex offers a higher power of discrimination than existing casework kits without impacting its performance. Compared to the YFiler Plus kit, the YFiler Platinum Casework kit can generate informative profiles with low DNA input and tolerates high levels of inhibition, such as 400 µM of hematin. No female artifacts above the peak amplitude threshold of 175 RFU were observed with up to 3 µg of co-amplified female DNA. In the two male mixture study, all non-overlapping alleles from the minor contributor were obtained with as low as 125 pg of male DNA.

In addition to the attributes mentioned above, several other characteristics are noteworthy—such as a faster PCR time of 60 min (86 min in the YFiler Plus kit) and the addition of an Internal Quality Control (IQC) system to indicate the success of the PCR amplification while discriminating between inhibition and degradation. The validation results presented here demonstrated high sensitivity, robust inhibitor tolerance, male specificity, concordance, and sizing accuracy and precision. These studies confirm that the YFiler Platinum Casework Kit meets its design requirements as a highly discriminating, reliable, and robust Y-STR analysis kit for forensic casework analysis.

## Data Availability

The Y-STR data presented in this manuscript has been uploaded to YHRD at https://yhrd.org/. The YHRD data will be uploaded into version 70. Accession number YA006024.
